# Common and Cluster-Specific Simultaneous Component Analysis

**DOI:** 10.1371/journal.pone.0062280

**Published:** 2013-05-08

**Authors:** Kim De Roover, Marieke E. Timmerman, Batja Mesquita, Eva Ceulemans

**Affiliations:** 1 Methodology of Educational Sciences Research Unit, KU Leuven, Leuven, Belgium; 2 Heymans Institute of Psychology, University of Groningen, Groningen, The Netherlands; 3 Social and Cultural Psychology Research Unit, KU Leuven, Leuven, Belgium; Georgetown University Medical Center, United States of America

## Abstract

In many fields of research, so-called ‘multiblock’ data are collected, i.e., data containing multivariate observations that are nested within higher-level research units (e.g., inhabitants of different countries). Each higher-level unit (e.g., country) then corresponds to a ‘data block’. For such data, it may be interesting to investigate the extent to which the correlation structure of the variables differs between the data blocks. More specifically, when capturing the correlation structure by means of component analysis, one may want to explore which components are common across all data blocks and which components differ across the data blocks. This paper presents a common and cluster-specific simultaneous component method which clusters the data blocks according to their correlation structure and allows for common and cluster-specific components. Model estimation and model selection procedures are described and simulation results validate their performance. Also, the method is applied to data from cross-cultural values research to illustrate its empirical value.

## Introduction

Researchers often gather data with a so-called ‘multiblock’ structure [1], i.e., multivariate observations nested within higher-level research units. The data then contain separate blocks of data, one for each higher-level research unit. These data blocks have the variable mode in common. For example, when a personality trait questionnaire is administered to inhabitants of different countries, the countries constitute the data blocks and the questionnaire items the variables. In case several emotions are measured multiple times for a number of subjects, the data blocks pertain to the different subjects and the variables to the emotions.

With such data at hand, it can be interesting to explore and summarize the correlation structure of the variables and possible between-block differences therein. For example, in the personality data mentioned above, one could look for cultural differences in the correlation structure of personality traits. Specifically, one could examine to what extent the well-known Big Five structure [2] is found within each country (e.g., [3]).

To capture such between-block differences in correlation structure, clusterwise simultaneous component analysis was recently proposed [4]–[5]. This method summarizes the most important structural differences and similarities by assigning the data blocks to a number of mutually exclusive clusters and, at the same time, performing a separate simultaneous component analysis (SCA) within each cluster. SCA generalizes standard PCA to multiblock data and models the different blocks using the same component loadings, whereas different restrictions can be imposed on the component scores to express similarities across blocks [6]. Hence, in Clusterwise SCA, data blocks with a similar structure are collected into the same cluster and thus modeled with the same loadings, while data blocks with different structures are allocated to different clusters. Thus, the differences in structures are expressed by differences in loadings across the clusters. For instance, a Clusterwise SCA analysis of the cross-cultural personality data would reveal which countries have a very similar personality trait structure by assigning those countries to the same cluster. Moreover, inspecting the loadings in the different clusters, one may gain insight into which part of the correlation structure differs across countries.

As clusters are modeled independently of one another, Clusterwise SCA may keep structural similarities across clusters hidden, however. Specifically, taking empirical results into account, it can often be assumed that some of the components are common across clusters, implying that the structural differences only pertain to a subset of the components. For instance, cross-cultural research on the Big Five has shown that three or four of the five components are found in all countries, whereas the interpretation of the fourth and fifth component can differ across countries [7]–[8].

Therefore, it may be useful to adopt a more flexible perspective on structural differences and similarities between clusters, where the differences can be situated somewhere along the length of a bipolar commonness dimension, of which the two poles pertain to commonness of all underlying components (implying that it is sufficient to use only one cluster and thus a regular SCA) on the one hand and no commonness on the other hand (implying that one should apply Clusterwise SCA). Between these poles we find models in which the structural differences between clusters concern only a subset of the underlying components, whereas the other components are common across clusters. For instance, as discussed above, for the cross-cultural personality data a model in which the clusters differ with respect to only one or two out of the five components and in which the other components are common, seems indicated. Hence, in this paper, we propose a method on the interface between regular SCA and Clusterwise SCA, to allow for common and cluster-specific components. More specifically, we propose to combine SCA-ECP and Clusterwise SCA-ECP (where ‘SCA-ECP’ refers to SCA with Equal Cross-Products constraints on the component scores) for simultaneously inducing the Common and Cluster-specific SCA-ECP components, respectively. This new method is named CC-SCA-ECP.

The remainder of the paper is organized in five sections: In the Method section, the CC-SCA-ECP model is introduced and compared to related methods, after a short discussion of the data structure and the recommended preprocessing. The Analysis section describes the loss function and an algorithm for performing a CC-SCA-ECP analysis, followed by a model selection heuristic. In the Simulation Study section, the performance of this algorithm and model selection heuristic is evaluated in a simulation study. In the Application section, CC-SCA-ECP is applied to cross-cultural data on values. Finally, we end with the Discussion section, including directions for future research.

## Methods

### 2.1. Data Structure and Preprocessing

CC-SCA-ECP is applicable to multiblock data, which are data that consist of *I* data blocks **X** *_i_* (*N_i_*×*J*) that contain scores of *N_i_* observations on *J* variables (measured at least at interval level). The number of observations *N_i_* (*i*  = 1, …, *I*) may differ between data blocks, subject to the restriction that *N_i_* is larger than the number of components to be fitted (and, to enable stable model estimates, preferably larger than *J*). The data blocks can be concatenated into an *N* (observations)×*J* (variables) data matrix **X**, where 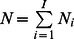.

With CC-SCA-ECP, we aim to model similarities and differences in the correlational structure of the different blocks (i.e., ‘within-block structure’). To achieve this, each variable is centered and standardized per data block prior to the CC-SCA-ECP analysis. This type of preprocessing is often referred to as ‘autoscaling’ [9] and is equivalent to calculating z-scores per variable within each data block. The centering step assures that one analyzes the within-block part of the data [10]. The standardizing step assures that one analyzes correlations. In multiblock analysis, next to autoscaling, other approaches are standardizing the variables across all data blocks (rather than per block) (e.g., [6]), or no scaling at all (e.g., [11]), with centering applied per data block. This implies that one models the within-block covariances rather than the correlations. A drawback of clustering the data blocks on the basis of their covariances, however, is that the obtained clustering may be based on variance differences as well as correlation differences, which complicates the cluster interpretation. Since we are exclusively interested in the correlational structure per block, we assume each data block **X** *_i_* to be autoscaled in what follows.

### 2.2. CC-SCA-ECP Model

To allow for common as well as cluster-specific components, CC-SCA-ECP combines an SCA-ECP model with a Clusterwise SCA-ECP model. Formally, data block **X** *_i_* is modeled as
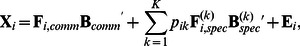
(1)with Equal Cross Product (ECP) constraints as 
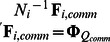
, 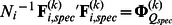, and with 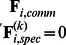 to ensure separation of common and specific components, for *i*  = 1, …, *I*. The first term of Equation 1 is the SCA-ECP model formula with 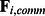 (*N_i_*×*Q_comm_*) containing the scores on the common components and 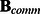 (*J*×*Q_comm_*) the loadings on the common components (see e.g., [6]). When a subgroup of variables has high loadings on a particular common component, this indicates that these variables are highly correlated in all data blocks and thus may reflect one underlying dimension (represented by the component). The entries of 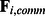 indicate how high or low the observations within the data blocks score on the common components. The second term is the Clusterwise SCA-ECP model formula where *K* denotes the number of clusters, with 1≤ *K* ≤ *I*, *p_ik_* is an entry of the partition matrix **P** (*I*×*K*) which equals one when data block *i* is assigned to cluster *k* and zero otherwise, and 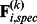 (*N_i_*×*Q_spec_*) and 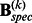 (*J*×*Q_spec_*) contain the scores and loadings on the cluster-specific components of cluster *k* (*k*  = 1, …, *K*). Finally, **E** *_i_* (*N_i_*×*J*) denotes the matrix of residuals. Note that we constrained the number of cluster-specific components *Q_spec_* to be the same for all clusters. The generalization toward a varying number of cluster-specific components 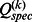 across clusters, which makes sense for some data sets, will be discussed later on (in the Discussion).

To gain more insight into what Equation 1 means for the decomposition of the total data matrix **X** (*N*×*J*), we rewrite it for an example where six data blocks are assigned to two clusters, and where the first four blocks belong the first cluster and the last two to the second cluster:
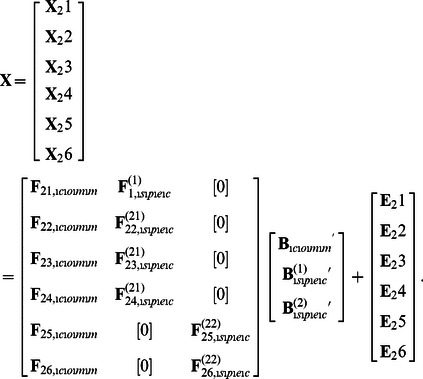
(2)


It can be seen that the data blocks have non-zero component scores on the common components and on the cluster-specific components of the cluster to which they are assigned, but zero scores on the cluster-specific components of the other cluster. As the clusters are mutually exclusive in terms of the data blocks they incorporate (i.e., each data block belongs to a single cluster only), this implies that the non-zero parts of the different cluster-specific columns cannot overlap.

The constraints imposed on the cross-products of common and cluster-specific components in CC-SCA-ECP can be represented as
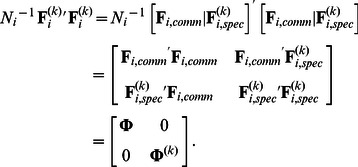
(3)


To identify the model, without loss of fit, we rescale the solution such that the diagonal elements of **Φ** and **Φ** ^(*k*)^ equal one. Furthermore, because the mean component scores for each data block **X** *_i_* equal zero (due to the centering of **X** *_i_* and the minimization function applied, see [6]), the matrices 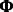 and 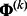 are correlation matrices of the common components and the cluster-specific components of cluster *k*, respectively. Note that we prefer to impose the orthogonality restrictions on the component scores rather than on the loadings, as this implies that (1) all restrictions pertain to the same parameters and (2) the loadings can be interpreted as correlations between the variables and the components.

Note that the common and cluster-specific components of a CC-SCA-ECP solution can be rotated without loss of fit, which can make them easier to interpret. Thus, 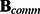 can be multiplied by any rotation matrix, provided that the corresponding component score matrices 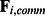 are counterrotated. Similarly, each 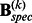 and the corresponding 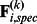 matrices (*k*  = 1, …, *K*) can be rotated.

In defining our model, we deliberately selected the most constrained variant of the SCA family [6], to obtain components that can unambiguously be interpreted as either common or cluster-specific. The use of a less constrained SCA variant, like SCA-IND, appears to be inappropriate for the following reasons. First, in that case a common component may have a variance of zero in a specific data block, which seriously undermines its common nature. Second, the clustering may sometimes be dominated by differences in component variances rather than by differences in correlational structure of the variables (see Section 2.1).

### 2.3. Relations to Existing Methods

In the literature, a few other techniques have been proposed for distinguishing between common components and components that underlie only part of the data blocks (e.g., GSVD; [12]–[15]). However, only two of them explicitly allow to extract a specified number of common and non-common components from multiblock data: DISCO-SCA [16]–[17] and OnPLS [18].

#### 2.3.1 DISCO-SCA

DISCO-SCA disentangles distinctive (‘DIS’) and common (‘CO’) components by rotating a regular SCA-P solution, where ‘P’ refers to the equal Pattern restriction, implying that no further restrictions are imposed on the component scores and thus that the component variances and component correlations may differ across blocks [6]. Specifically, the DISCO-SCA rotation criterion minimizes the variance explained in data block *i* by a component that is distinctive for other data blocks but not for data block *i*. Thus, a distinctive component is defined as a component that explains a negligible amount of variance in some of the data blocks. Schouteden, Van Deun and Van Mechelen [19] also proposed DISCO-GSCA, which adapts DISCO-SCA in that it not only tries to maximize the ‘distinctiveness’ of distinctive components but also imposes it to a certain degree (implying some loss of fit).

DISCO-SCA and CC-SCA-ECP differ essentially in their definitions of common and non-common components: In DISCO-SCA, non-common (i.e., distinctive) components are obtained by explicitly looking for components that explain as little variance as possible in the data blocks for which they are not distinctive, without loss of fit. As the component scores of this distinctive component are not explicitly restricted to zero in the other data block(s), a common and a distinctive component can be correlated within a certain data block, however. In our view, the interpretation of such distinctive components is rather intricate, because they may carry common information. In contrast, in CC-SCA-ECP non-common (i.e., specific) components merely have a different loading pattern in the different clusters. Specifically, CC-SCA-ECP maximizes the variance explained by the common and specific components under the restriction that the common and specific components are orthogonal within each data block.

Note that the zero scores in Equation 2 might suggest that the specific components of a particular cluster cannot explain any variance in any other cluster. This is not generally true, however. If we would start from a given CC-SCA-ECP solution, and would extend the solution by freely estimating the component scores that are associated with zeros in Equation 2, the fit of the extended model would probably be larger than the CC-SCA-ECP model itself, and thus the cluster-specific components can model some of the variance in other clusters. This implies that those components are not truly distinctive in DISCO-SCA terms.

Furthermore, DISCO-SCA is limited to finding common and distinctive components within the SCA-P subspace (for DISCO-GSCA this is only partly the case) and is therefore biased toward finding common components as such components often will explain the most variance in the data. CC-SCA-ECP is less restrictive in this respect, because the cluster-specific components are estimated separately for each cluster and thus only need to explain enough variance within these clusters to be retrieved (under the restriction that they are orthogonal to the common components).

Finally, CC-SCA-ECP can easily handle a large number of data blocks by means of the clustering, while DISCO-(G)SCA was originally intended for the analysis of two data blocks. Although the general idea behind DISCO-(G)SCA can be extended to more than two data blocks [20], it will soon become complex since components can be distinctive for all conceivable subsets of the data blocks implying that many degrees of distinctiveness become possible.

#### 2.3.2 OnPLS

OnPLS [18] is another method that aims to distinguish between common and specific variance. This method was developed for object-wise linked multiblock data, but it can be applied to variable-wise linked multiblock data by simply considering the transposed data matrices 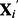 for all *i*. OnPLS starts by computing an orthogonalized version of each data block in which the shared or common variance with the other data blocks is removed. Next, specific components are obtained for each data block by finding those components that optimally summarize the associated orthogonalized data block. Finally, the common components are extracted from the residual matrices, which are obtained by subtracting the specific parts (captured by the specific components) from the original data blocks, with all common and specific components being orthogonal. An important difference between the common components resulting from OnPLS and the common components in DISCO-(G)SCA and CC-SCA-ECP is that the OnPLS components model variance that is common across data blocks, but that the scores or loadings are not constrained to be the same across all data blocks. On top of that, OnPLS differs from CC-SCA-ECP in three respects. First, OnPLS uses a sequential approach in that it first extracts the specific components from the data set and then models the common variance, while CC-SCA-ECP uses a simultaneous approach for finding the clustering and the common and cluster-specific components. Second, unlike CC-SCA-ECP, OnPLS imposes that the specific components of different data blocks are orthogonal. Third, like DISCO-(G)SCA, OnPLS does not include a clustering of the data blocks and will thus be less insightful than CC-SCA-ECP in case of a large number of data blocks.

## Analysis

### 3.1. Aim

For a given number of clusters *K*, number of common components *Q_comm_* and number of cluster-specific components *Q_spec_*, the aim of the analysis is to find the partition matrix **P**, the component score matrices 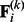 = 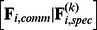 and the loading matrices 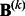
_ = 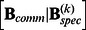,_ that minimize the loss function
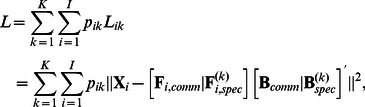
(4)subject to the constraints formulated in Equation 3. Based on the loss function value *L*, one can compute the percentage of variance in the data that is accounted for by the CC-SCA-ECP solution:



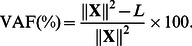
(5)Note that this VAF(%) can be further decomposed into the percentage of variance that is explained by the common part:
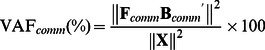
(6)and the percentage of variance that is explained by the cluster-specific part: 
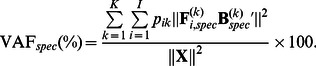
(7)


### 3.2. Algorithm

In order to find the solution with the minimal *L* value (Equation 4), an alternating least squares (ALS) algorithm is used, in which the partition, component scores and loading matrices are updated cyclically until convergence is reached. As ALS algorithms may converge to a local minimum, we recommend to use a multistart procedure and retain the best solution. More specifically, we advise to use a ‘rational’ start based on a Clusterwise SCA-ECP analysis and several (e.g., 25) random starts. For each start, the algorithm performs the following steps:

1. Initialize partition matrix **P**: For a rational start, take the best partition resulting from a Clusterwise SCA-ECP analysis with *K* clusters and *Q_comm_*+*Q_spec_* components (performed with 25 random starts, as advised by De Roover et al. [4]). For a random start, assign the *I* data blocks randomly to one of the *K* clusters, where each cluster has an equal probability of being assigned to and each cluster should contain at least one data block.
2. Estimate common and cluster-specific components, given partition matrix **P**: To this end, another ALS procedure is used, consisting of the following steps:
  - Initialize loading matrices **B***_comm_* and 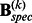: **B** *_comm_* and **F** *_comm_* are initialized by performing a rationally started SCA-ECP analysis with *Q_comm_* components on the total data matrix **X** (for more details, see [6]). Next, the cluster-specific loadings 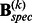 and component scores 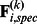 for cluster *k* are obtained by performing a rationally started SCA-ECP with *Q_spec_* components on 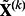, which is the vertical concatenation of the data blocks in the *k*-th cluster after subtracting the part of the data that is modeled by the common components (i.e., 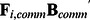) from each block in that cluster.
  - Update the component score matrices 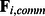 and 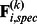: To obtain orthogonality of 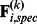 toward_
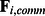
_ for each data block (see Section 2.2), the component scores of the *i*-th data block (in cluster *k*) are updated as 
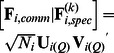
, where **U** *_i_*, **V** *_i_* and **S** *_i_* result from a singular value decomposition 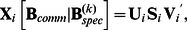 and where **U** *_i_*_(*Q*)_ and **V** *_i_*_(*Q*)_ are the first *Q* columns of **U** *_i_* and **V** *_i_* respectively, and **S** *_i_*_(*Q*)_ consists of the first *Q* rows and columns of **S** *_i_*, with *Q* equal to *Q_comm_*+*Q_spec_*. Note that, compared to Equation 3, this updating step implies additional orthogonality constraints, i.e., all columns of the obtained 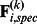 and_
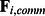
_ are orthogonal, but this can be imposed without loss of generality.
  - Update the loadings **B***_comm_* and 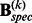: **B** *_comm_* is re-estimated by 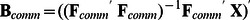 and 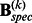 is updated per cluster by 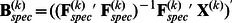, where 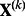 and 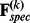 are the vertical concatenations of the data blocks that are assigned to cluster *k* and of their component scores 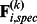, respectively.
  - Alternate steps b and c until convergence is reached, i.e., until the decrease of the loss function value *L* (Equation 4) for the current iteration is smaller than the convergence criterion, which is 1×10^−6^ by default.
3. Update the partition matrix **P**: Each data block **X** *_i_* is tentatively assigned to each of the *K* clusters. Based on the loading matrix 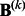, a component score matrix for block *i* in cluster *k* is computed (as in step 2b) and the loss function value *L_ik_* (see Equation 4) of data block *i* in cluster *k* is evaluated. The data block is assigned to the cluster for which this loss is minimal. If one of the clusters is empty after this step, the data block with the worst fit in its current cluster, is reassigned to the empty cluster.
4. Repeat steps 2 and 3 until the partition **P** no longer changes. In this procedure, the common loadings **B** *_comm_* and component scores **F** *_comm_* of the previous iteration are used as a start for step 2, instead of the rational start described in step 2a, since a change in the partition will primarily affect the cluster-specific components.

Because the clustering of each block in step 3 is based on the loadings resulting from step 2 (i.e., the loadings are not updated after each reassignment; it is possible to update the common and cluster-specific loadings for each reassignment, but we chose not to because this would strongly inflate the computation time), it cannot be guaranteed that this algorithm monotically non-increases the loss function. However, we did not encounter problems in this regard, neither for the simulated data sets (Simulation Study section) nor for the empirical example (Application section).

### 3.3. Model Selection

In empirical practice, theoretical knowledge can lead to an a priori expectation about the number of clusters *K*, the total number of components *Q* (i.e., *Q_comm_*+*Q_spec_*) and/or the number of cluster-specific components *Q_spec_* that is needed to adequately describe a certain data set. For instance, when exploring the underlying structure of cross-cultural personality trait data, one probably expects five components based on the Big Five theory [2], of which one or two may be cluster-specific. However, when one has no expectations about *K*, *Q* and/or *Q_spec_*, a model selection problem arises. To offer some assistance in dealing with this problem, the following CC-SCA-ECP model selection procedure is proposed, which is based on the well-known scree test [21]:

1. Estimate Clusterwise SCA-ECP models with one to *K*^max^ clusters and one to *Q*^max^ components within the clusters: *K* ^max^ and *Q* ^max^ are the maximum number of clusters and components one wants to consider. Note that in this step, all components are considered to be cluster-specific.
2. Obtain *K*^best^ and *Q*^best^: To select among the *K* ^max^×*Q* ^max^ models from step 1, De Roover, Ceulemans and Timmerman [1] proposed the following procedure: First, to determine the best number of clusters *K* ^best^, scree ratios *sr*_(*K*|*Q*)_ are calculated for each value of *K*, given different *Q*-values:
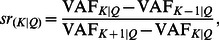
(8)where VAFK_|Q_ indicates the VAF(%) (Equation 5) of the solution with K clusters and Q components. The scree ratios indicate the extent to which the increase in fit with additional clusters levels off; therefore, K^best^ is chosen as the K-value with the highest average scree ratio across the different Q-values. Second, scree ratios are calculated for each value of Q, given K^best^ clusters:

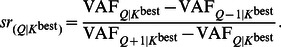
(9)The best number of components *Q* ^best^ is again indicated by the maximal scree ratio.

1. Estimate all possible CC-SCA-ECP models with *K*^best^ clusters and *Q*^best^ components: Perform CC-SCA-ECP analyses with *K* ^best^ clusters and with one to *Q* ^best^ cluster-specific components per cluster and the rest of the *Q* ^best^ components considered common.
2. Select 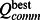 and 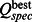: Given *K* ^best^ clusters and *Q* ^best^ components, a CC-SCA-ECP model becomes more complex as more of the *Q* ^best^ components are considered cluster-specific. Consequently, a scree test is performed with the number of cluster-specific components *Q_spec_* as a complexity measure. Specifically, the following scree ratio is computed for each *Q_spec_*-value:

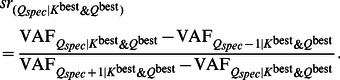
(10)The maximum scree ratio will indicate the best number of cluster-specific components 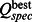 and thus also the best number of common components 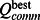 (i.e., *Q* ^best^ – 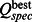). When *Q* ^best^ is smaller than four, it makes no sense to perform a scree test; in those cases, we recommend to compare the models estimated in step 3 with respect to explained variance and interpretability.

When a priori knowledge is available about *K* or *Q*, this knowledge can be applied within steps 1 and 2. If *K* and *Q* are both known, steps 1 and 2 can be skipped. Note that, even though the above-described procedure will retain only one model as ‘the best’ for a given data set, we advise to take the interpretability of the other solutions with high scree ratios into account, especially when the results of the model selection procedure are not very convincing. For instance, when the maximum scree ratio for the number of components (Equation 10) is only slightly higher than the second highest scree ratio (thus corresponding to the second best number of components *Q* ^2ndbest^), one can also apply steps 3 and 4 for solutions with *Q* ^2ndbest^ components and consider both the best solution with *Q* ^best^ components and with *Q* ^2ndbest^ components and eventually retain the one that gives the most interpretable results.

## Simulation Study

### 4.1. Problem

In this simulation study, we evaluate the performance of the proposed algorithm with respect to finding the optimal solution (i.e., avoiding local minima) and recovering the underlying model (i.e., the correct clustering of the data blocks and the correct common and cluster-specific loadings). Moreover, we examine whether the presented model selection procedure succeeds in retaining the correct model (i.e., the correct number of clusters, common components, and cluster-specific components). Specifically, we assess the influence of six factors: (1) number of underlying clusters, (2) number of common and cluster-specific components, (3) cluster size, (4) amount of error on the data, (5) amount of common (structural) variance, and (6) similarity or congruence of the cluster-specific component loadings across clusters. The first four factors are often varied in simulation studies to evaluate clustering or component analysis algorithms (see e.g., [22]–[28]), and were also examined in the original Clusterwise SCA-ECP simulation study [4]. With respect to these factors, we expect better model estimation and selection results when less clusters and components are involved [4], [23]–[25], [27], when the clusters are of equal size [22], [24], [26], and when the data contain less error [4], [23], [28]. Regarding Factors 5 and 6, we conjecture that model estimation and selection will deteriorate when the amount of common variance increases and when the cluster-specific component loadings are more similar across clusters.

Moreover, one might conjecture that CC-SCA-ECP does not add much to Clusterwise SCA-ECP, because one could just apply Clusterwise SCA-ECP and examine whether one or more of the components are strongly congruent across clusters and are thus essentially common. We see two possible pitfalls, however: (a) estimating all components as cluster-specific can affect the recovery of the clustering in case the data contain a lot of error and the truly cluster-specific components explain little variance; (b) strong congruence between components across clusters can be hard to detect, even when the components of different clusters are rotated toward maximal congruence [29], because Clusterwise SCA-ECP will partly model the cluster-specific error and therefore hide the commonness of some components. To gain insight into the differences between Clusterwise SCA-ECP and CC-SCA-ECP, we will compare the performance of both methods.

### 4.2. Design and Procedure

Fixing the number of variables *J* at 12 and the number of data blocks *I* at 40 (note that lowering the number of data blocks to 20 yields very similar results), the six factors introduced above were systematically varied in a complete factorial design:

1. the *number of clusters K* at 2 levels: 2, 4;
2. the *number of common and cluster-specific components Q_comm_ and Q_spec_* at 4 levels: [*Q_comm_*, *Q_spec_*] equal to [2,2]; [2], [3]; [3], [2]; [3,3];
3. the *cluster size*, at 3 levels (see [24]): equal (equal number of data blocks in each cluster); unequal with minority (10% of the data blocks in one cluster and the remaining data blocks distributed equally over the other clusters); unequal with majority (60% of the data blocks in one cluster and the remaining data blocks distributed equally over the other clusters);
4. the *error level e*, which is the expected proportion of error variance in the data blocks **X** *_i_*, at 2 levels:.20,.40;
5. the *common variance c*, which is the expected proportion of the structural variance (i.e., 1– *e*) that is accounted for by the common components, at 3 levels:.25,.50,.75;
6. the *congruence between the cluster-specific component loadings* at 2 levels: low congruence, medium congruence.

For each cell of the factorial design, 20 data matrices **X** were generated, consisting of 40 **X** *_i_* data blocks. The number of observations for each data block was sampled from a uniform distribution between 30 and 70. The entries of the component score and error matrices **F** *_i_* and **E** *_i_* were randomly sampled from a standard normal distribution. The partition matrix **P** was generated by computing the size of the different clusters and randomly assigning a corresponding number of data blocks to the clusters. The cluster loading matrices 
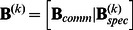
 were created by sampling the common loadings **B** *_comm_* uniformly between –1 and 1 and rescaling their rows to have a sum of squares equal to the amount of common variance *c*. The cluster-specific loading matrices 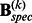 with low congruence were obtained in the same way, but their rows were rescaled to have a sum of squares equal to the amount of cluster-specific variance (1– *c*). Cluster-specific loading matrices with medium congruence were constructed as follows: (1) a common base matrix and *K* specific matrices were uniformly sampled between –1 and 1, (2) the rows of these matrices were rescaled to have a sum of squares equal to.7*(1– *c*) and.3*(1– *c*), respectively, (3) the *K* specific matrices were added to the base matrix. To evaluate how much the resulting cluster-specific loading matrices differ between the clusters, they were orthogonally procrustes rotated to each other (i.e., for each pair of cluster-specific loading matrices, one was chosen to be the target matrix and the other was rotated toward the target matrix) and a congruence coefficient 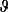 [30] was computed – the congruence coefficient for a pair of column vectors **x** and **y** is defined as their normalized inner product: 
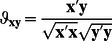
 – for each pair of corresponding components in all pairs of 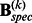 matrices. Subsequently, a grand mean of the obtained 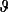 values was calculated, over the components and cluster pairs. Since Haven and ten Berge [31] demonstrated that congruence values from.70 to.85 correspond to an intermediate similarity between components, only 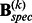 matrices with a mean 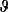 below.70 were retained for the low congruence level and loading matrices with a mean 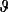 between.70 and.85 for the medium congruence level. Eventually, averaging the mean 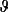 values across the simulated data sets led to an average 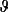 of.39 (*SD*  = 0.09) for the low congruence level and an average 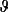 of.78 (*SD*  = 0.04) for the medium congruence level. Next, the error matrices **E** *_i_* and the cluster loading matrices 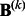 were rescaled to obtain the correct amount of error variance *e*. Finally, the resulting **X** *_i_* matrices were standardized per variable and vertically concatenated into the matrix **X**.

In total, 2 (number of clusters)×4 (number of common and cluster-specific components)×3 (cluster size)×3 (common variance)×2 (congruence of cluster-specific components)×2 (error level)×20 (replicates)  = 5,760 simulated data matrices were generated. For the model estimation part of the simulation study, each data matrix **X** was analyzed with the CC-SCA-ECP algorithm, using the correct number of clusters *K*, and the correct numbers of common and cluster-specific components *Q_comm_* and *Q_spec_*. The algorithm was run 26 times, using one rational start and 25 different random starts (see Section 3.2), and the best solution was retained. These analyses took about 16 minutes per data set on a supercomputer consisting of INTEL XEON L5420 processors with a clock frequency of 2.5 GHz and with 8 GB RAM. Additionally, a Clusterwise SCA-ECP analysis with 25 random starts was performed for each data matrix.

The model selection part of the simulation study is confined to the first five replications of each cell of the design to keep the computational cost within reasonable limits. For each of these 1,440 data matrices, the stepwise model selection procedure (see Section 3.3) was performed with *K* ^max^ equal to six and *Q* ^max^ equal to seven.

### 4.3. Results

#### 4.3.1. Model estimation

We will first discuss the sensitivity to local minima (given that *K*, *Q_comm_* and *Q_spec_* are known), followed by an evaluation of the goodness-of-recovery of the clustering of the data blocks and the loadings of the common and cluster-specific components. Finally, we will compare the performance of Clusterwise SCA-ECP and CC-SCA-ECP.

To evaluate the sensitivity of the CC-SCA-ECP algorithm to local minima, the loss function value of the retained solution should be compared to that of the global minimum. This global minimum is unknown, however, because the simulated data are perturbed with error and because, due to sampling fluctuations, the data do not perfectly comply with the CC-SCA-ECP assumptions (e.g., the orthogonality constraints on the common and cluster-specific components). As a way out, we use the solution that results from seeding the algorithm with the true **F** *_i_*, **B** ^(*k*)^ and **P** matrices as a proxy of the global minimum.

Specifically, we evaluated whether the best fitting solution out of the 26 runs (i.e., one rational and 25 random starts) had a higher loss function value than the proxy, which would imply that this solution is a local minimum for sure. The latter is the case for 345 out of the 5,760 data sets (6%). Note that this number is a lower bound of the true number of local minima (which cannot be determined because the global minimum is unknown). The majority of the established local minima (i.e., 326 out of the 345) occur in the conditions with unequal cluster sizes, with a medium congruence between the cluster-specific components, and/or with the cluster-specific components accounting for only 25% of the structural variance. Using only the rational start would have resulted in 1,178 (20%) local minima and using only the 25 random starts in 576 (10%) local minima, thus combining rational and random starts seems necessary to keep the sensitivity to local minima sufficiently low.

To examine how well the cluster memberships of the data blocks are recovered, the Adjusted Rand Index (*ARI*; [32]) is computed between the true partition of the data blocks and the estimated one. The *ARI* equals one if the two partitions are identical, and equals zero when the agreement between the partitions is at chance level. With an overall mean *ARI* of.98 (*SD*  = 0.11) the CC-SCA-ECP algorithm appears to recover the clustering of the data blocks very well. More specifically, an incorrect clustering (i.e., *ARI* <1.00) occurred for only 415 out of the 5,760 data sets. The majority of these clustering mistakes (i.e., 414 out of the 415) occurred in the most difficult conditions, i.e., 75% common structural variance combined with 40% error variance.

Furthermore, to assess the extent to which the goodness-of-recovery of the clustering deteriorates in case of local minima, we took a closer look at the *ARI* values for the 345 data sets for which we obtained a local minimum for sure. The mean *ARI* for these 345 data sets amounts to.81 (*SD*  = 0.28), which is clearly lower than the overall mean. Surprisingly, the clustering is still recovered perfectly (i.e., *ARI*  = 1.00) for 192 of these data sets. For the remaining 153 data sets, according to the guidelines reported by Steinley [33], the cluster recovery is excellent (*ARI* between.90 and 1.00) for 20, good (*ARI* between.80 and.90) for 27, moderate (*ARI* between.65 and.80) for 18 and bad (i.e., *ARI* <.65) for 88 of these data sets. Thus, for the majority of the local minima, the clustering is still good to excellent.

To evaluate the goodness-of-recovery of the loadings of the common components, we calculated a goodness-of-common-loading-recovery statistic (*GOLR_comm_*) by computing congruence coefficients 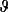 [30] between the common components of the true and estimated loading matrices and averaging these coefficients across the *Q_comm_* components:
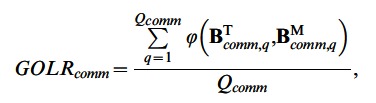
(11)with 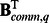 and 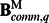 indicating the *q*-th common component of the true and estimated loading matrices, respectively. To deal with the rotational freedom of the common components, the estimated common components were orthogonally procrustes rotated toward the true ones. The *GOLR_comm_* statistic takes values between zero (no recovery at all) and one (perfect recovery), and – according to Lorenzo-Seva & ten Berge [34] – two components can be considered identical when their congruence coefficient is above.95. On average, the *GOLR_comm_* has a value of.99 (*SD*  = 0.02), indicating an excellent recovery of the common loadings. Moreover, the *GOLR_comm_* is smaller than.95 for only 146 out of the 5,760 data sets, of which 137 belong to the conditions with 40% error variance.

For the 345 data sets that are confirmed local minima, the mean *GOLR_comm_* is equal to.98 (*SD*  = 0.04). Furthermore, the recovery of the common loadings is excellent (*GOLR_comm_* >.95) for 315 of these data sets. Therefore, we can conclude that the common loadings are still recovered very well for the majority of the local minima.

For quantifying the recovery of the cluster-specific component loadings, we compute a statistic similar to the *GOLR_comm_*, namely as
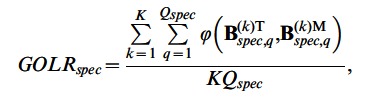
(12)where 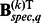
and 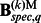 refer to the *q*-th component of the true and estimated cluster-specific loading matrices, respectively, and averaging across the *Q_spec_* components and the *K* clusters. The rotational freedom of the components was again dealt with by orthogonal procrustes rotations. Moreover, the true and estimated clusters were matched such that the *GOLR_spec_* value is maximized.

The *GOLR_spec_* value amounts to.98 on average (*SD*  = 0.04) and is higher than.95 for 5,207 out of the 5,760 data sets, showing that also the cluster-specific components are recovered very well. Out of the 553 data sets for which *GOLR_spec_* is smaller than.95, 435 are situated in the conditions with 75% common structural variance and 40% error variance, which are conditions wherein the cluster-specific variance (i.e., (1– *c*)×(1– *e*)  = .15) is strongly masked by the error variance (i.e., *e*  = .40).

For the 345 confirmed local minima, the mean *GOLR_spec_* is equal to.92 (*SD*  = 0.10). The cluster-specific loadings are recovered excellently (*GOLR_spec_* >.95) for 205 of these data sets, which is still more than half of them. Thus, the recovery of the cluster-specific loadings is affected by the fact that these solutions are local minima, but even then they seem to be recovered quite well.

To compare the performance of CC-SCA-ECP and Clusterwise SCA-ECP, we first evaluate whether the clustering obtained with CC-SCA-ECP is closer to the true clustering (i.e., higher *ARI*) than that resulting from a Clusterwise SCA-ECP analysis with *Q_comm_*+*Q_spec_* components. This is the case for 330 out of the 5,760 data sets (6%), with an average *ARI* improvement of.18 and with perfect CC-SCA-ECP cluster recovery (i.e., *ARI*  = 1.00) for 122 of these data sets. All of the 330 data sets are situated in the – difficult – conditions with 75% common variance and/or 40% error variance. Conversely, the Clusterwise SCA-ECP *ARI* was better than the CC-SCA-ECP *ARI* for 94 data sets only (with an average *ARI* gain of.11, but with the Clusterwise SCA-ECP *ARI* remaining smaller than 1.00 for 77 out of these 94 data sets). Of these data sets, 93 were situated in the conditions with 75% common variance and/or 40% error variance, which are the hardest clustering conditions. Second, we examine to what extent the common components could be traced in the Clusterwise SCA solution. To this end, for each data set, we computed the following mean between-cluster congruence coefficient:
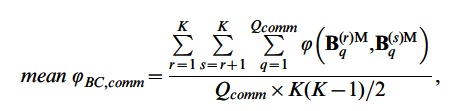
(13)where the subset of *Q_comm_* components is used that yields the highest 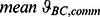 coefficient and where_
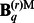
_ and 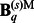 are the *q*-th components of clusters *r* and *s*. Of course, the 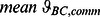 values could depend on how the rotational freedom of the components is dealt with. Therefore, we calculated the 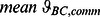 values for three different rotational approaches. First, we adopted the practice that was advocated in the Clusterwise SCA papers [4], [5], i.e., rotating each cluster loading matrix toward simple structure, for which we make use of the varimax criterion. We took the permutational freedom of the components into account by retaining the permutation of the components in cluster *s* that maximizes the mean congruence with the components in cluster *r*. Second, we used procrustes rotation which is available in some statistical software and thus is an obvious candidate to explore the presence of common components. Specifically, we orthogonally procrustes rotated the components of the second up to the *K*-th cluster toward the varimax rotated components of the first cluster. The first cluster thus serves as the reference cluster and the loadings of this reference cluster are rotated toward simple structure to obtain a better interpretability. Which cluster is chosen as reference may have some influence on the obtained 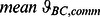 value. Third, we included the rotation procedure that is expected to give the best results in revealing commonness of components, that is, rotating the components of the *K* clusters toward maximal congruence by means of the method presented by Brokken [35] and adapted by Kiers and Groenen [29].

On average, the 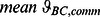 amounts to.81 (*SD*  = 0.11),.88 (*SD*  = 0.09) and.93 (*SD*  = 0.05) for the varimax, procrustes and maximal congruence rotation, respectively. To quantify how often the most similar components across the clusters can actually be interpreted as equal and therefore common components, we employ the guidelines reported by Lorenzo-Seva and ten Berge [34], who state that components can be considered equal in terms of interpretation when their congruence is.95 or higher. For the varimax rotation approach, the between-cluster congruence of the common components is below.95 for no less than 5,512 (96%) out of the 5,760 data sets, while for the procrustes rotation the 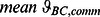 is below.95 for 4,439 (77%) of the simulated data sets. Even for the rotation toward maximal congruence, the 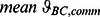 is below.95 for 2,877 (50%) of the data sets. In other words, for a large proportion of the simulated data sets, applying Clusterwise SCA-ECP rather than CC-SCA-ECP would be inappropriate, because it cannot be revealed that some of the components are shared by all clusters.

#### 4.3.2. Model selection

The model selection procedure described in Section 3.3 selects the correct model – i.e., the correct number of clusters, the correct number of common components, and the correct number of cluster-specific components – for 648 or about 45% of the 1,440 simulated data sets included in the model selection study. If we investigate these results into more detail, we see that the number of clusters is correctly assessed for 1,223 or 85% of the data sets, which is reasonable given the error levels of 20% and 40%. Out of the 217 mistakes, 213 are made when the cluster-specific variance amounts to only 25% of the structural variance and/or when the cluster-specific components are moderately congruent, which makes sense as the underlying clusters are harder to distinguish in these conditions.

The total number of components *Q* is correct for only 771 or 54% of the simulated cases, however. Out of the 669 mistakes, 645 are made in the conditions with 40% error variance and/or with a low amount of common or cluster-specific variance. This result is explained by the fact that (some of) the common components may be considered minor in case of 25% common variance and (some of) the cluster-specific components may turn out to be minor in case of 75% common variance, especially when the data contain a lot of error. Therefore, the total number of components is often underestimated as the scree test is known to focus more on the major components [36]–[37]. Also, minor components occur more often when the number of components is relatively high, as is the case in our study. Indeed, many model selection studies on component analysis techniques have shown that the performance decreases when more components are involved (e.g., [38]–[39]).

As the selection of the number of common components *Q_comm_* and the number of cluster-specific components *Q_spec_* strongly depends on whether or not the correct total number of components is retained, it is no surprise that *Q_comm_* and *Q_spec_* are selected correctly in only 52% and 63% of the cases, respectively. Indeed, if we exclusively take the 771 data sets into account for which the total number of components is correct, the correct *Q_spec_*, and thus also the correct *Q_comm_*, is selected in 93% of the cases, which is excellent.

Finally, in the above paragraphs, we discussed the results obtained when both the number of clusters and the number of components have to be estimated. However, in practice, often some a priori knowledge is available, considerably simplifying the model selection problem. Therefore, we end this section by investigating what happens if the total number of components is known beforehand. In this case, the number of clusters is selected correctly for 1,306 or 91% of the data sets, and the numbers of common and cluster-specific components in 1,271 or 88% of the data sets. Out of the 169 mistakes against *Q_spec_*, 159 are situated in conditions with medium congruence among cluster-specific component structures and/or with six components, implying underestimation of the number of cluster-specific components. Finally, if we look at the number of clusters as well as the numbers of common and cluster-specific components, when the total number of components is known, both are selected correctly for 82% of the data sets (i.e., for 1,183 out of the 1,440 data sets).

Based on these simulation results, we can formulate some guidelines for CC-SCA-ECP model selection in empirical practice. As stated before, using prior knowledge on the expected number of clusters and/or components may be advantageous. When such knowledge is not available, one can apply the model selection procedure described in Section 3.3, but scrutinize the suggested number of components *Q*. Indeed, as befits a scree test procedure, *Q* is often underestimated when one or more of the common or cluster-specific components is minor. Therefore, when in doubt on how many components should be retained (e.g., the scree ratio for the selected *Q*-value is only slightly higher than that for another *Q*-value), one should also consider solutions with the *Q*-values indicated by the second – the second best number of components is the correct one for 20% of the data sets in the simulation study; thus, in 74% of the simulated cases the correct number of components is among the two best *Q*-values – and third highest scree ratio [27] and perform steps 3 and 4 of the model selection procedure for these *Q*-values as well. Consequently, one ends up with two or three CC-SCA-ECP solutions from which the best one can be chosen based on interpretability.

## Application

To illustrate the empirical value of CC-SCA-ECP, we will apply it to cross-cultural data on values from the International College Survey (ICS) 2001 [40]–[41]. Up to now, most research in this domain (e.g., [42]–[43]) focuses on the mean score of inhabitants of particular countries on broader value dimensions. We take a different approach as we will examine the correlation structure of a set of specific values within countries and model between-country similarities and differences therein.

The ICS study included 10,018 participants out of 48 different nations. Each of them rated, among other things, how much they valued eleven aspects, which are listed in Table 1, using a 9-point likert scale (1 =  “do not value it at all”, 9 =  “value it extremely”). 330 participants with missing data were excluded. Differences between the countries in the means and the variances of the values were removed by standardizing the values per country (see Section 2.1).

**Table 1:** Common and cluster-specific loadings of the CC-SCA-ECP model with two clusters, one common and one cluster-specific component for the value data from the 2001 ICS study.

	Common	Cluster-specific	
		Cluster 1	Cluster 2
	General value dimension	Happiness & achievement	Fun & intelligence vs. showing success
Happiness	**.52**	**.49**	.22
Intelligence/knowledge	**.39**	**.58**	**.65**
Material wealth	**.63**	−.22	−**.40**
Physical attractiveness	**.68**	−.25	−**.43**
Physical comforts	**.65**	−.08	−**.44**
Excitement/arousal	**.61**	−.24	−**.35**
Competition	**.61**	−.15	−**.40**
Heaven/afterlife	**.44**	**.48**	−**.42**
Self-sacrifice	**.47**	.25	−.28
Success	**.61**	**.55**	.33
Fun	**.35**	.18	**.70**

Loadings greater than +/−. 35 are highlighted in boldface.

**Figure 1:**
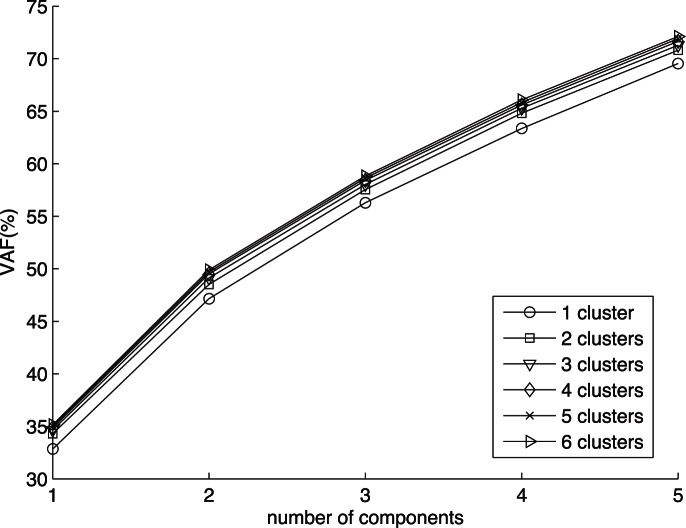
Percentage of explained variance of the Clusterwise SCA-ECP solutions for the value data from the 2001 ICS study, with the number of cluster varying from one to six, and the number of components varying from one to five.

To find an optimal CC-SCA-ECP model for these data, we used the model selection procedure described in Section 3.3. We first performed Clusterwise SCA-ECP analyses with one to six clusters and one to five components within each cluster. In Figure 1 the VAF(%) of the obtained Clusterwise SCA-ECP solutions is plotted against the number of components for each number of clusters. In step 2 of the model selection procedure the model with two clusters and two components per cluster is retained as the best Clusterwise SCA-ECP model, because the mean of the scree ratios 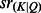 (Equation 8) is highest for two clusters and, given two clusters, the scree ratio 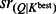 (Equation 9) for the number of components is maximal for two components (see Table 2). Indeed, the data are fitted considerably better using two clusters rather than one, while adding extra clusters hardly improves the fit, and, regarding the number of components, the increase in fit with extra components levels off after two components (see Figure 1).

**Table 2:** Scree ratios 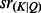 for the numbers of clusters *K* given the numbers of components *Q* and averaged over the numbers of components (above), and scree ratios 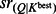 for the numbers of components *Q* given two clusters (below), for the value data of the 2001 ICS study.

	1 comp	2 comp	3 comp	4 comp	5 comp	average
2 clusters	**4.12**	**2.22**	**2.53**	**3.16**	**2.66**	**2.94**
3 clusters	1.78	1.60	1.30	1.48	1.30	1.49
4 clusters	1.17	2.12	1.66	1.35	1.46	1.55
5 clusters	1.27	0.95	1.16	0.94	1.20	1.10
	2 clusters					
2 components	**1.58**					
3 components	1.24					
4 components	1.21					

The maximal scree ratio in each column is highlighted in boldface.

**Figure 2:**
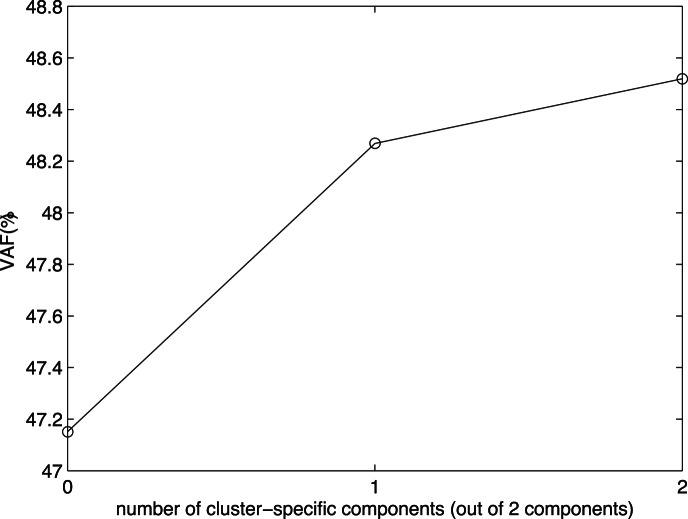
Percentage of explained variance plotted against the number of cluster-specific components for (from left to right) SCA-ECP with two components (i.e., both components common), CC-SCA-ECP with one common and one cluster-specific component and Clusterwise SCA-ECP with two clusters and two components (i.e., both components cluster-specific), for the value data from the 2001 ICS study.

We determine how many of these components can be taken as common by performing CC-SCA-ECP with one common and one cluster-specific component and comparing the VAF(%) of this solution with that of the Clusterwise SCA-ECP solution with two (cluster-specific) components and that of the SCA-ECP solution with two (common) components. As Figure 2 shows, allowing one of the components to be cluster-specific gives a considerable increase in fit, while making the second component cluster-specific adds very little. Because the model with one cluster-specific component and one common component is more parsimonious than the model with two cluster-specific components, while the fit is about equal, we select the model with one common and one cluster-specific component.

Upon inspecting the clustering given in Table 3, we can conclude that cluster 1 contains the African, South, Southeast, and West Asian countries (note that Cyprus and Georgia are often classified as West Asian countries [44]), with the exception of Nepal and Zimbabwe. Cluster 2 contains the other 33 countries.

**Table 3:** Clustering of the countries of the CC-SCA-ECP model for the value data from the 2001 ICS study, with two clusters, one common and one cluster-specific component.

Cluster 1	Bangladesh, Cameroon, Cyprus, Egypt, Georgia, Ghana, Indonesia, Iran, Kuwait, Malaysia, Nigeria, Philippines, South Africa, Thailand, Uganda
Cluster 2	Australia, Austria, Belgium, Brazil, Bulgaria, Canada, Chile, China, Colombia, Croatia, Germany, Greece, Hong Kong, Hungary, India, Italy, Japan, Mexico, Nepal, Netherlands, Poland, Portugal, Russia, Singapore, Slovakia, Slovenia, South Korea, Spain, Switzerland, Turkey, United States, Venezuela, Zimbabwe

The common and cluster-specific loadings are shown in Table 1. Note that, since we have only one common and one cluster-specific component, there is no rotational freedom. All values load strongly on the common component, which accounts for 30% of the variance in the data, implying that this component can be interpreted as a general value dimension indicating that in each of the 48 countries all values are positively correlated. The cluster-specific component of cluster 1, which explains 13% of the associated variance, is labeled ‘Happiness & achievement’ as it displays high positive loadings of ‘Happiness’, ‘Intelligence and knowledge’, ‘Getting to heaven, achieving a happy afterlife’ and ‘Success’. The cluster-specific component of cluster 2, which explains 20% of the corresponding variance, has a very different loading pattern. Specifically, it has highly positive loadings of ‘Intelligence and knowledge’ and ‘Fun (personal enjoyment)’, and highly negative loadings of ‘Material wealth’, ‘Physical attractiveness’, ‘Physical comforts’, ‘Excitement and arousal’, ‘Competition’ and ‘Getting to heaven, achieving a happy afterlife’. Therefore, we named this component ‘Fun & intelligence versus showing success’. In these countries some people mainly pursue intelligence and fun in their lives (e.g., look for a job that offers many opportunities to develop abilities and grants a lot of satisfaction), while some of the others mainly value showing his or her success in life (e.g., look for a job with a high salary or a high status).

To gain more insight into these between-country differences in within-country correlation structure (modeled by the cluster-specific component), we tried to map them onto two value dimensions that distinguish between cultures with different levels of modernization [43]–[44]. Firstly, we focused on the traditional versus secular-rational dimension, that distinguishes values that are dominant in pre-industrial societies from those of industrial ones: In comparison to secular-rational countries, traditional countries emphasize religion and respect for (parental) authority, male dominance in economic and political life, and national pride. Secondly, we used the survival versus self-expression dimension that disentangles traditional/industrial societies (stronger focus on economic and physical security) and post-industrial ones (stronger focus on self-expression and quality of life).

**Figure 3:**
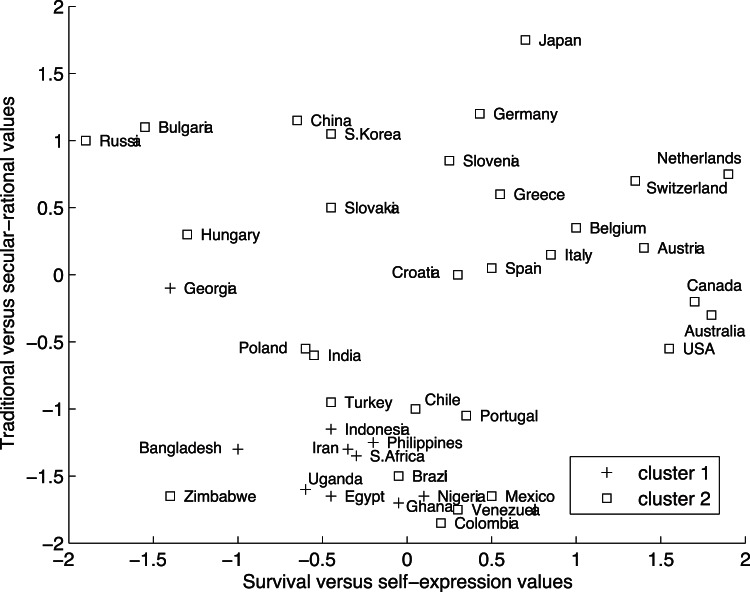
Reproduction of the cultural values map published by Inglehart and Welzel [44], retaining only the countries that are included in the ICS study and indicating to which cluster each country belongs in the CC-SCA-ECP model for the ICS values data.

To relate the cross-cultural differences with respect to these dimensions to the differences found by CC-SCA-ECP, Figure 3 reproduces the cultural values map published by Inglehart and Welzel [44], only retaining the countries included in the ICS study. Note that Figure 3 contains only 40 out of the 48 countries included in the ICS study, because Inglehart and Welzel [44] did not report mean scores for the other eight countries. From this figure, it is clear that cluster 1 contains pre-industrial countries scoring low on both dimensions (with the exception of Zimbabwe), while the other countries are gathered in cluster 2. This suggests that participants from pre-industrial countries that are both more traditional and more focused on the basic values necessary for survival tend to (more or less) pursue both happiness and achievement (i.e., intelligence and knowledge, getting to heaven, success) together. Participants from countries which are more secular-rational and/or more focused on self-expression either pursue intelligence and fun (i.e, mental rewards) or strive to show off their success (i.e., material or interpersonal rewards).

Finally, to illustrate the added value of CC-SCA-ECP over Clusterwise SCA-ECP, we will comment on the Clusterwise SCA-ECP solution with two components in each cluster. The clustering of this solution is nearly identical to the clustering of the CC-SCA-ECP solution, with the difference that Egypt is assigned to cluster 2 instead of to cluster 1. This is inconsistent with our finding that the countries scoring low on both dimensions in Figure 3 are gathered in cluster 1. Furthermore, even when the components of the clusters are rotated toward maximal congruence [29], the Tucker phi coefficients of the most similar components amount to.82 and.93, implying that none of them can be considered identical. This conclusion is further supported by the considerable differences between the maximal congruence loadings of both clusters (e.g., the loadings of fun on the first component differ in sign), that are shown in Table 4. Thus, this rotation strategy does not disentangle the common from the cluster-specific component.

**Table 4:** Maximal congruence rotated loadings of the Clusterwise SCA-ECP model with two clusters and two components per cluster for the value data from the 2001 ICS study.

	Cluster 1		Cluster 2	
	Fun & showing success	Fun, happiness, achievement & benevolence	Fun vs. showingsuccess & benevolence	Fun, happiness &achievement
Happiness	.06	**.73**	.12	**.56**
Intelligence/knowledge	−.05	**.73**	−.32	**.69**
Material wealth	**.65**	.30	**.67**	.26
Physical attractiveness	**.71**	.31	**.73**	.28
Physical comforts	**.58**	**.43**	**.72**	.25
Excitement/arousal	**.67**	.29	**.63**	.26
Competition	**.64**	.27	**.67**	.27
Heaven/afterlife	.02	**.66**	**.59**	.12
Self-sacrifice	.23	**.53**	**.49**	.20
Success	.07	**.78**	.09	**.71**
Fun	**.36**	**.51**	−**.41**	**.61**

Loadings greater than +/−. 35 are highlighted in boldface.

## Discussion

This paper introduced the CC-SCA-ECP method for multiblock data, which can be used to study between-block similarities and differences in correlation structure. Specifically, CC-SCA-ECP combines the key features of SCA-ECP and Clusterwise SCA-ECP by clustering the data blocks under study and by distinguishing common components that underlie all data blocks and cluster-specific components that reveal between-block differences. Because of the implied clustering, CC-SCA-ECP is especially useful when the number of data blocks becomes somewhat larger (say, larger than five) as the other available methods for multiblock data, DISCO-SCA and OnPLS, cannot easily handle such a larger number.

In the presence of common components, CC-SCA-ECP was shown to outperform Clusterwise SCA-ECP in two important respects: First, in the more difficult simulation conditions, CC-SCA-ECP often yielded a better clustering than Clusterwise SCA-ECP. Similarly, the obtained CC-SCA-ECP clustering in our empirical example was more consistent with known differences between countries than the Clusterwise SCA-ECP clustering. Second, for more than half of the simulated data sets as well as the empirical example, it proved impossible to rotate the obtained Clusterwise SCA-ECP components in such a way that commonness of some of the components could be detected.

At this point, we want to emphasize the added value of CC-SCA-ECP in comparison to multigroup factor analysis methods [45]–[47], which are commonly used to test different levels of measurement invariance among the data blocks (see [48] for more details). Where measurement invariance tests merely indicate whether the factor structures (and, in case of strict invariance, also the intercepts and unique variances) are the same across all data blocks or not, CC-SCA-ECP actually explores what the structural differences are. Specifically, on the one hand, it looks for subgroups of data blocks with an identical structure and, on the other hand, it captures which subset of the components is different between these subgroups (i.e., clusters).

The differences between CC-SCA-ECP on the one hand and DISCO-SCA and OnPLS on the other hand imply some points of discussion and possible directions for future research. First, in Section 2.3, we argued that the CC-SCA-ECP cluster-specific components are not necessarily distinctive between clusters in the sense that they may explain some variance in the other clusters if the zero restrictions in Equation 2 would be removed. Although this may seem a disadvantage at first sight, the mere ‘specificity’ of the cluster-specific components actually makes the method more versatile. Indeed, CC-SCA-ECP may reveal subtle differences in the functioning of a few variables. This can, for instance, be very interesting when assessing the measurement invariance of a particular questionnaire in different groups. Moreover, if the data contain truly distinctive components, these will easily be picked up by CC-SCA-ECP. Nonetheless, it may be interesting to develop a CC-SCA-ECP variant that, to some extent, imposes distinctiveness on the cluster-specific components (i.e., they should not explain a lot of variance in the other clusters). The latter might, for instance, be achieved by adding a penalty term to the loss function that takes into account how well data blocks in one cluster can be reconstructed by cluster-specific components of other clusters.

Second, taking the different degrees of distinctiveness (e.g., one component can be distinctive for data block 1 and 2, another for data blocks 3 to 5, and so on) that are possible in DISCO-SCA into account, it may be useful to extend CC-SCA-ECP to incorporate different degrees of cluster-specificity (i.e., components can be specific for more than one cluster, implying that they are shared or common for these clusters, but not for others).

Third, one might consider it too strict to require the common and cluster-specific components to be orthogonal in each data block. Indeed, it might occur for some data sets that one of the cluster-specific components is correlated with a common component in one or more of the data blocks, which is an important structural aspect that cannot be captured by CC-SCA-ECP. We want to emphasize, however, that, next to the obvious technical advantages, the orthogonality restriction has an important substantive advantage in that it prevents the method from finding cluster-specific components that are nearly a copy of the common components.

Fourth, we restricted the number of cluster-specific components 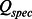 to be the same for each cluster, which might be unrealistic for some data sets. Indeed, Clusterwise SCA-ECP has been generalized to allow the number of components to vary over clusters [49], so extending this approach toward CC-SCA-ECP seems straightforward. While this generalization is feasible with respect to model estimation (the algorithm has been developed and can be obtained from the first author), it would make model selection, which already proved to be very challenging in the current paper, even more intricate. Therefore, we propose to use a post-hoc strategy. Specifically, one may consider to let the number of CC-SCA-ECP cluster-specific components differ across clusters when a Clusterwise SCA-ECP solution with a varying number of components contains components that are very similar among clusters and can therefore be conceived as common, or when some of the CC-SCA-ECP cluster-specific components indicate overextraction (e.g., a component with only one high loading, a meaningful subgroup of variables seems to be arbitrarily divided over two components, etc.).

Finally, we have never tested the applicability of the CC-SCA-ECP method to high-dimensional data, so it is not that easy to predict how the method will behave in those cases. Therefore, it would be useful for future research to test the performance of CC-SCA-ECP for simulated high-dimensional data. We suspect that the method will fail when these data contain a lot of noise and the cluster-specific components are moderately or strongly congruent across clusters.
