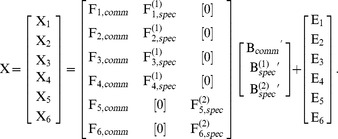# Correction: Common and Cluster-Specific Simultaneous Component Analysis

**DOI:** 10.1371/journal.pone.0093796

**Published:** 2014-04-01

**Authors:** The PLOS ONE Staff

In the Methods section, there are errors in the first and second equation in the section titled "2.2. CC-SCA-ECP Model." The publisher apologizes for these errors. The second line after the first equation incorrectly describes the Equal Cross Product (ECP) constraints. The correct equation is:
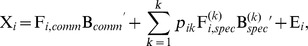



with Equal Cross Product (ECP) constraints as 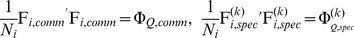, and with 
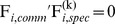
 to ensure separation of common and specific components[…]

There is an additional error in the second equation, many numbers were added. The correct equation is: